# Ferroptosis contributes to multiple sclerosis and its pharmacological targeting suppresses experimental disease progression

**DOI:** 10.1038/s41418-023-01195-0

**Published:** 2023-08-04

**Authors:** Emily Van San, Angela C. Debruyne, Geraldine Veeckmans, Yulia Y. Tyurina, Vladimir A. Tyurin, Hao Zheng, Sze Men Choi, Koen Augustyns, Geert van Loo, Bernhard Michalke, Vivek Venkataramani, Shinya Toyokuni, Hülya Bayir, Peter Vandenabeele, Behrouz Hassannia, Tom Vanden Berghe

**Affiliations:** 1 Department of Biomedical Molecular Biology, Ghent university, Ghent, Belgium; 2 VIB-UGent Center for Inflammation Research, Ghent, Belgium; 3 Department of Human Structure and Repair, Ghent University, Ghent, Belgium; 4 Department of Biomedical Sciences, University of Antwerp, Antwerp, Belgium; 5 Department of Environmental Health and Occupational Health, University of Pittsburgh, Pittsburgh, PA USA; 6 Department of Pathology and Biological Responses, Nagoya University Graduate School of Medicine, Nagoya, Japan; 7 Department of Pharmaceutical Sciences, University of Antwerp, Antwerp, Belgium; 8 Research Unit Analytical BioGeoChemistry, Helmholtz Zentrum München, Munich, Germany; 9 Department of Pathology, University Medical Center, Goettingen, Germany; 10 Center for Low-temperature Plasma Sciences, Nagoya University, Furo-cho, Chikusa, Nagoya, Japan; 11 Children’s Neuroscience Institute, UPMC Children’s Hospital of Pittsburgh, Pittsburgh, PA USA; 12 Methusalem program, Ghent University, Ghent, Belgium

**Keywords:** Neurological disorders, Translational research, Drug development, Experimental models of disease

## Abstract

Multiple sclerosis (MS) is a chronic autoimmune disorder characterized by central nervous (CNS) demyelination resulting in axonal injury and neurological deficits. Essentially, MS is driven by an auto-amplifying mechanism of inflammation and cell death. Current therapies mainly focus on disease modification by immunosuppression, while no treatment specifically focuses on controlling cell death injury. Here, we report that ferroptosis, an iron-catalyzed mode of regulated cell death (RCD), contributes to MS disease progression. Active and chronic MS lesions and cerebrospinal fluid (CSF) of MS patients revealed several signs of ferroptosis, reflected by the presence of elevated levels of (labile) iron, peroxidized phospholipids and lipid degradation products. Treatment with our candidate lead ferroptosis inhibitor, UAMC-3203, strongly delays relapse and ameliorates disease progression in a preclinical model of relapsing-remitting MS. In conclusion, the results identify ferroptosis as a detrimental and targetable factor in MS. These findings create novel treatment options for MS patients, along with current immunosuppressive strategies.

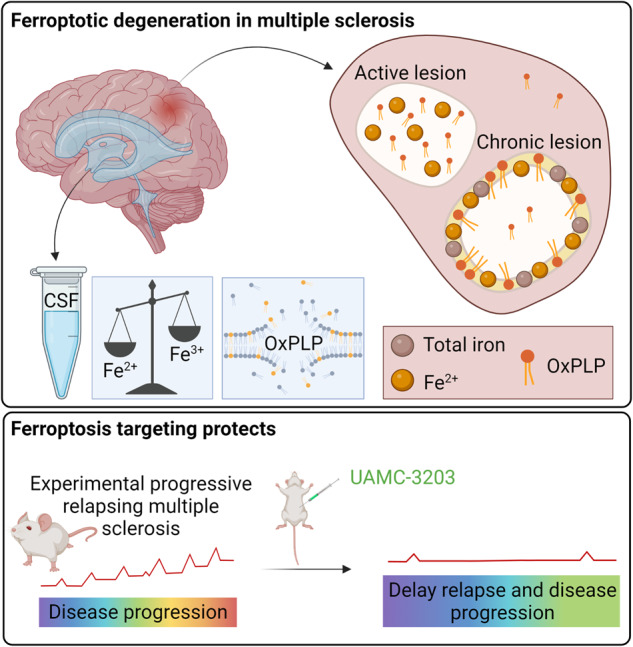

## Introduction

Multiple sclerosis (MS) is the most common cause of non-traumatic neurological injury affecting quality of life, physical functioning, and cognitive performance. Worldwide a total of 2.8 million people (35.9 per 100,000 population) are estimated to be affected with MS [1]. Current treatment strategies for MS, especially relapsing-remitting (RRMS) and secondary progressive MS (SPMS), are mainly based on immunosuppressive disease-modifying therapies, to reduce the rate of relapses and lower short-term deterioration [2]. Although the auto-amplifying mechanism between inflammation and cell death in MS pathology is generally recognized, no cell death-targeting therapies have made it to the clinic yet. Several mediators of apoptosis, pyroptosis, and necroptosis have been proposed to contribute to progressive neurodegeneration in experimental MS models [3–8], arguing for the involvement of multiple regulated cell death modalities (RCD) in this complex and heterogeneous pathology. Essentially, progressive neurodegeneration is mainly due to the death of neurons and oligodendrocytes [9]. Given the high content of iron in these cells, dysregulated iron homeostasis is known to contribute to neurodegenerative diseases such as MS [10]. Labile iron is highly reactive and able to catalyze the formation of phospholipid peroxyl radicals causing cellular disruption [11], a process coined as ferroptosis [12]. Phospholipids containing polyunsaturated fatty acids (PUFAs) are very susceptible to peroxidation, contributing to membrane permeabilization and cell lysis [13, 14]. The inner phospholipid layer of the myelin sheath (composed of oligodendrocyte membrane wraps) is enriched by PUFAs [15], suggesting that oligodendrocytes might be susceptible to ferroptosis. It is predicted that the presence of PUFAs in the myelin sheath may destabilize the dense structure in diseased conditions [16]. Therefore, we hypothesized that ferroptosis might be a detrimental factor in MS pathology.

Cell death by ferroptosis is genetically prevented by the lipid repair enzyme glutathione peroxidase 4 (GPX4), which converts lipid hydroperoxides to unreactive lipid alcohols [17]. Glutathione (GSH) is a crucial antioxidant and is required for the proper functioning of GPX4. It has already been shown that GSH levels are reduced in the cerebrospinal fluid (CSF) of MS patients and that GPX4 activity is affected during MS pathology [18]. Recently, several key genetic factors have been identified to be crucial in controlling ferroptosis such as GTP cyclohydrolase 1 (GCH1) [19], ferroptosis suppressor protein (FSP1) [20], and dihydroorotate dehydrogenase (DHODH) [21]. Ferroptosis can be prevented by using iron chelators, natural lipophilic radical traps such as vitamin E as well as synthetic radical traps such as ferrostatin-1 (Fer1) and liproxstatin-1 (Lip1) [22–24].

In this report, we reveal a ferroptosis signature in MS patient samples, reflected by elevated levels of labile iron, peroxidized phospholipids, and lipid degradation products, such as 4-hydroxy-2-nonenal (4-HNE). We investigated the therapeutic potential of ferroptosis targeting in a clinically relevant experimental model for relapsing-remitting (RRMS) and SPMS in Biozzi ABH mice [25] and found that inhibition of ferroptosis ameliorates disease progression and strongly delays relapse. This finding identifies ferroptosis as an important contributor to MS pathology and demonstrates that ferroptosis targeting might be a promising novel future treatment for MS patients, eventually supplementing current immunosuppressive disease-modifying therapies.

## Results

### Active and chronic MS lesions show ferroptosis signatures

**Fig. 1:**
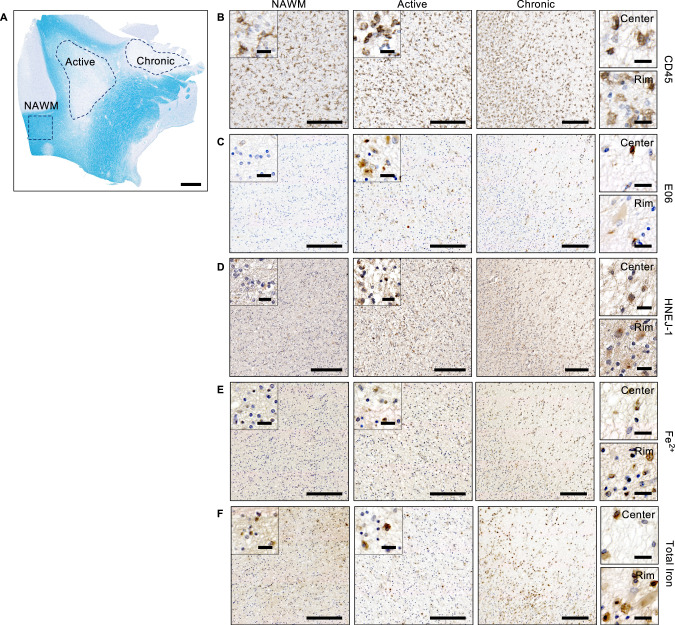
Active and chronic MS lesions show increase in inflammation, 4-HNE adducts, oxPC deposition and ferrous iron. **A** Representative LFB/PAS-stained brain tissue indicating NAWM, active and chronic region (demarcated by blue dotted line) from a MS patient sample. Scale bar, 2 mm. **B**–**F** Histopathological analysis of NAWM, active and chronic region of MS patient. Scale bar, 200 µm and zoom section, 20 µm. NAWM, active and chronic region show CD45^+^ microglia throughout lesion. CD45^+^ myeloid cells and amoeboid microglia are predominantly present in active and center of chronic lesion, while rim is mainly characterized by CD45^+^ foamy microglia (**B**). Signatures of lipid peroxidation, HNEJ-1 and E06, are increased in active and throughout chronic lesion, compared to nearly absence of lipid peroxidation in NAWM (**C**, **D**). Ferrous iron is increased throughout active and chronic lesion (**E**), while total non-heme iron drops in active and center of chronic lesion and an increase is observed at border of chronic lesion compared to NAWM (**F**) (in total *n* = 4 patient, see Supplementary Figs. S1–6, for representative images of 3 additional patients). MS multiple sclerosis, HNE 4-hydroxyl-2-nonenal, oxPC oxidized phosphatidylcholines, LFB Luxol fast blue, PAS periodic acid Schiff, NAWM normal-appearing white matter.

Approximately 85–90% of patients are diagnosed with RRMS, of which 80% develop SPMS and 10–15% develop primary progressive MS (PPMS). Both SPMS and PPMS patients show comparable lesion activity and no differences in lesion pathology [26]. Therefore, we selected brain tissue based on presence of different lesion types independent of disease course. We first investigated the possible association between ferroptosis signatures and MS pathology on paraffin-embedded brain tissue from SPMS patients [26]. To this end, we compared regions with and without lesions, referred to as respectively MS versus MS control samples. As non-MS controls, samples from non-demented patients were selected (Table S1). One region of normal-appearing white matter (NAWM), one active lesion, and one mixed active/inactive (chronic) lesion were selected in each MS patient sample (*n* = 4) for further immunohistopathological analysis (Fig. 1A and S1). NAWM was mainly characterized by the presence of ramified microglia and the center of chronic lesions show typical presence of CD45^+^ myeloid cells, amoeboid and foamy microglia, as reported previously [26, 27] (Fig. 1B and S2). The chronic rim predominantly consists of foamy microglia (Fig. 1B and S2A).

Ferroptosis is a phospholipid peroxidation-driven mode of regulated cell death [11]. To assess the extent of lipid peroxidation, sections were stained either for the presence of oxidized phosphatidylcholines (E06) [28] or 4-HNE adducts (HNEJ-1) [29]. High immunoreactivity for both E06 and 4-HNE was observed in active and chronic lesions, indicating excessive lipid peroxidation in MS lesions (Fig. 1C, D, S3A and S4A). In particular, the highest signal for lipid peroxidation was observed in active lesions followed by the rim and some positive signal in the center of chronic lesions (Fig. 1C, D, S3A and S4A). Only a few cells with oxidized phosphatidylcholines or 4-HNE modified proteins were found in NAWM of MS and non-MS tissue (Fig. 1C, D, Fig. S3 and S4). Another key regulator of the ferroptosis process is iron, especially redox-active ferrous iron (Fe^2+^), which is able to initiate and/or accelerate the generation of phospholipid hydroperoxides [30, 31]. We observed an increase in Fe^2+^ in active MS lesions and in the rim of chronic lesions compared to NAWM and non-MS controls (Fig. 1E and S5). In contrast to the chronic rim, Fe^2+^ containing cells were less abundant in the lesion center. Total non-heme iron was lower in active and the center of chronic lesions compared to NAWM, while also an increase was observed at the rim of chronic lesions (Fig. 1F and S6), which is in line with a previous report [32]. In summary, both active and chronic MS lesions show ferroptotic features including increase in labile iron, the presence of peroxidized phospholipids, and an accumulation of lipid degradation products.

### Ferroptosis signature in cerebrospinal fluid of MS patients

**Fig. 2:**
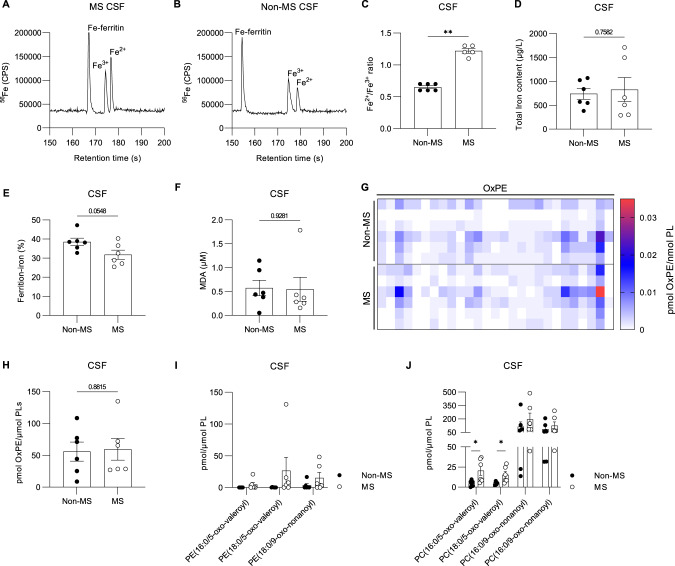
Elevated Fe^2+^/Fe^3+^ ratio and oxidized phosphatidylcholines species in CSF of MS patients. Representative electropherogram of iron redox and ferritin bound iron speciation analysis in MS CSF sample (**A**) and non-MS CSF sample (**B**), Fe^2+^/Fe^3+^ ratio is significantly increased in CSF of MS patients (**C**), while no significant difference is observed for total iron content (**D**), nor ferritin bound iron (**E**). Excessive lipid peroxidation is not detected by MDA levels (**F**), nor by screening for OxPE species represented in heat map showing amounts of OxPE in CSF of non-MS and MS patients (**G**) and corresponding quantification (**H**). Levels of truncated PE (**I**) and PC were analyzed indicating elevated levels of PC(16:0/5-oxo-valeroyl) and PC(18:0/5-oxo-valeroyl) in CSF of MS patients (**J**). Data is represented as mean ± SEM. Data was analyzed using a two-tailed Mann–Whitney U test (**C**, **F**) or by a two-tailed unpaired *t*-test **(D**, **E**, **H**–**J**) (*p ≤ 0.05 **p ≤ 0.01). CSF cerebrospinal fluid, MS multiple sclerosis, MDA malondialdehyde, OxPE oxidized phosphatidylethanolamine, PE phosphatidylethanolamine, PC phosphatidylcholine.

Next, we analyzed the CSF of MS and non-demented control patients for the presence of ferroptosis signatures (Table S1). Specification of total iron content, ferrous, ferric, and ferritin-bound iron was done by capillary electrophoresis coupled plasma mass spectrometry (CE-ICP-DRC-MS) [33], which resulted in high-quality electropherograms for both MS-derived CSF (Fig. 2A) and non-MS CSF samples (Fig. 2B). We found a significant increase in the ratio of ferrous iron versus ferric iron (Fe^2+^/Fe^3+^) in CSF of MS patients (Fig. 2C), which might be associated with the observed increase in Fe^2+^ in active and chronic MS lesions (Fig. 1E). The total iron content (Fig. 2D) and ferritin-bound iron (Fig. 2D) were similar when comparing non-MS and MS-affected CSF. Related to excessive lipid peroxidation, we didn’t detect an increase in malondialdehyde (MDA), end-products of lipid peroxidation, or peroxidized phosphatidylethanolamines (OxPE), nor OxPE truncated products, in CSF from MS patients (Fig. 2F–I). However, in line with the increased signal of OxPC in MS lesions (Fig. 1A and S3), elevated levels of oxidized truncated phosphatidylcholines (PC), PC(16:0/5-oxo-valeroyl) and PC(18:0/5-oxo-valeroyl), were observed in CSF of MS patients compared to non-MS CSF (Fig. 2J). Thus, an increased Fe^2+^/Fe^3+^ ratio and elevated levels of oxidized truncated phosphatidylcholines in CSF associate well with the ferroptotic signature observed in MS lesions. This may be further examined for the prognosis of disease progression.

### Immune response waves during experimental relapsing-remitting MS

**Fig. 3:**
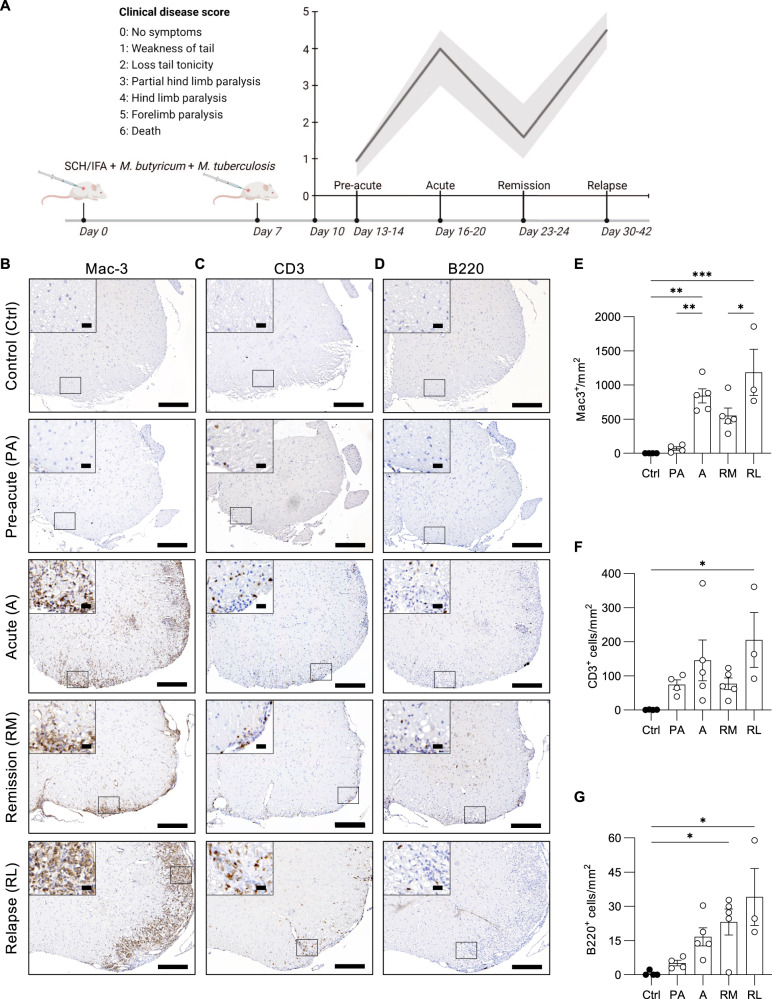
Macrophage and T cell infiltration correlates with clinical disease score during experimental RRMS, while B cells progressively increase. **A** Experimental autoimmune encephalomyelitis (EAE) induction in Biozzi ABH mice. Biozzi ABH mice were immunized subcutaneously with SCH in IFA, supplemented with *M. butyricum* and *M. tuberculosis*, on day 0 and day 7 as described in material and method section. Clinical scoring of disease started at day 10 and mice were sacrificed between day 13-14 (pre-acute, PA), day 16-20 (acute, A), day 23-24 (remission, RM) and day 30-42 (relapse, RL) to collect specimens in a kinetic manner. Unimmunized mice were used as healthy control (Ctrl). **B–D** Immunohistochemistry on lumbar spinal cord sections isolated from Ctrl (*n* = 4), PA (*n* = 4), A (*n* = 5), RM (*n* = 5) and RL (*n* = 3) EAE Biozzi ABH mice. Scale bar, 200 µm and zoom section, 20 µm. **B** Staining for infiltrating macrophages (Mac-3), T cells (CD3) (**C**), B cells (B220) (**D**), and corresponding quantification **E**–**G**, respectively. Data is represented as mean ± SEM. Data was analyzed using one-way ANOVA with Tukey’s correction for multiple comparisons (**E, F**), or with Kruskal–Wallis test followed by Dunn’s multiple comparisons test (**G**) (*p ≤ 0.05, **p ≤ 0.01, ***p ≤ 0.001). SCH spinal cord homogenate, IFA incomplete Freund’s adjuvant.

To study the therapeutic potential of targeting ferroptosis in MS, an RRMS model was set up in Biozzi ABH mice [25]. Depending on the clinical disease score and body weight, mice were sacrificed during the pre-acute, acute, remitting, and relapsing phase (Fig. 3A). In mice, induction of experimental autoimmune encephalomyelitis (EAE) results in inflammation and injury mainly in the spinal cord instead of the brain or optic nerves [34]. To examine the influx of immune cells, histopathological analysis was performed on lumbar spinal cord sections of mice, either or not immunized with spinal cord homogenate, by labeling for Mac-3^+^ macrophages, B220^+^ B cells, and CD3^+^ T cells. Infiltration of Mac-3^+^ macrophages significantly increases during the acute phase and relapse, when compared to non-immunized control littermates (Fig. 3B, E). Similarly, CD3^+^ T cells are detected during acute disease, seem to drop during remission, and significantly increase again during relapse (Fig. 3C, F), while B220^+^ B cells (Fig. 3D, G) continuously accumulate over time (PA, A, RM, RL) and only become significantly apparent during remission. Conclusively, these data reveal the acute immune attacks by macrophages and T cells during relapses, whereas chronic progression of the disease is associated with B cell infiltration.

### Cell death waves during experimental relapse remitting MS

**Fig. 4:**
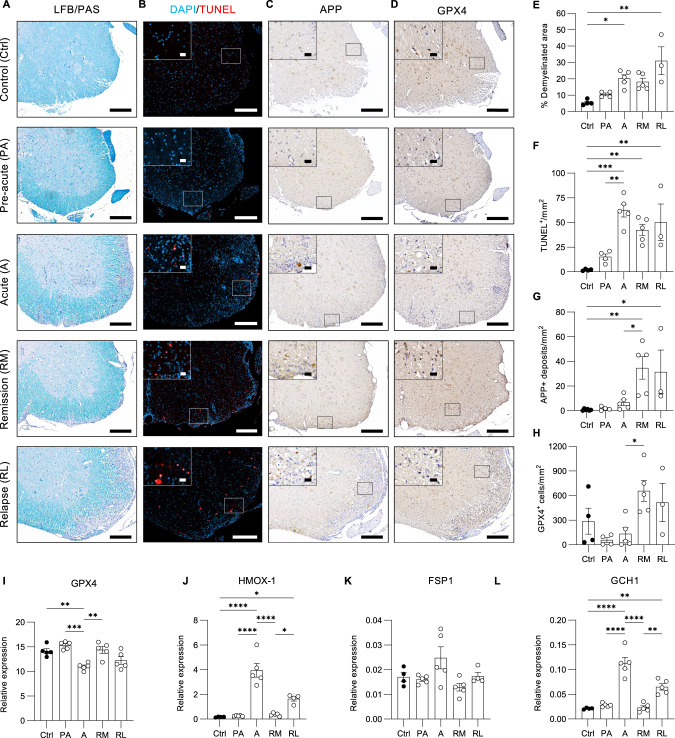
Cell death with transcriptional ferroptosis signature correlates with clinical disease score during experimental RRMS, while demyelination progressively increases. (Immuno)histochemistry on lumbar spinal cord sections isolated from Ctrl (*n* = 4), PA (*n* = 4), AC (*n* = 5), RM (*n* = 5) and RL (*n* = 3) EAE Biozzi ABH mice. Scale bar, 200 µm and zoom section, 20 µm. Assessment and quantification of demyelination by LFB/PAS staining (**A**, **C**), cell death by TUNEL (**B**, **F**), axonal damage by APP (**C**, **G**) and GPX4 expressing cells (**D**, **H**). Relative expression of ferroptosis associated factors GPX4 (**I**), HMOX-1 (**J**), FSP1 (**K**) and GCH1 (**L**) in spinal cord of Ctrl (*n* = 4-5), PA (*n* = 5), AC (*n* = 5), RM (*n* = 5) and RL (*n* = 4-5) EAE Biozzi ABH mice. Data is represented as mean ± SEM (**E**–**H**) or shown as ratio of mRNA expression normalized to endogenous housekeeping genes and expressed as mean ± SEM (**I**–**L**). Data were analyzed using one-way ANOVA with Tukey’s correction for multiple comparisons (**F**–**L**), or with Kruskal–Wallis test followed by Dunn’s multiple comparisons test (**E**) (*p ≤ 0.05, **p ≤ 0.01, ***p ≤ 0.001, ****p ≤ 0.0001). EAE experimental autoimmune encephalomyelitis, LFB Luxol fast blue, PAS Periodic acid Schiff, APP amyloid-β precursor protein, GPX4 glutathione peroxidase 4, HMOX-1 Heme oxygenase 1, FSP1 ferroptosis suppressor protein 1, GCH1 GTP cyclohydrase-1.

To characterize the extent of injury over time in experimental RRMS, Luxol fast blue (LFB)/PAS staining was performed on spinal cord sections to examine white matter (WM) demyelination (Fig. 4A). Compared to healthy controls, a significant increase in demyelinated area was observed during the acute phase of the disease and even aggravated during relapse (Fig. 4E), reflecting progressive demyelination. Dying TUNEL^+^ cells first appear during the pre-acute phase, but increase significantly at the acute phase and only slightly decrease during remission (Fig. 4B, F). Axonal damage, evaluated by the presence of amyloid precursor protein (APP^+^) deposits, firstly appears at remission (Fig. 4C, G), as previously reported [35]. As one of the hallmarks of ferroptosis, GPX4 levels (Fig. 4D, H, I) were reduced during the acute phase followed by a strong increase during remission (likely reflecting a recovery feedback mechanism). Note also an overall decrease in neuronal GPX4 expression in spinal cord grey matter (Fig. 4D), as previously reported [36]. Reversely, we observed a strong transcriptional increase in heme oxygenase-1 (HMOX1) during the acute phase and relapse (Fig. 4J). This protective response might be an attempt to reduce the levels of oxidative stress but alternatively might accelerate ferroptosis in case of excessive HMOX-1 activation [30]. In addition, FSP1 and GCH1, two other markers of ferroptosis [19, 20], are also transcriptionally upregulated during acute disease, possibly counteracting the loss of GPX4 (Fig. 4K, L). Also prostaglandin-endoperoxide synthase 2 (PTGS2) and heat shock protein beta-1 (HSPB1) were increased during the acute and relapse phase of the disease, but dropped during remission (Fig. S7A, B), further providing evidence of ferroptosis involvement in relapsing-remitting EAE. Transferrin receptor 1 (TFR1) slightly increases during pre-acute EAE, but significantly drops while progressing to the first relapse (Fig. S7C). In summary, cell death with a transcriptional signature of ferroptosis could be demonstrated during acute disease and relapse, while demyelination progressively increases over time.

### Ferroptosis targeting ameliorates experimental RRMS

**Fig. 5:**
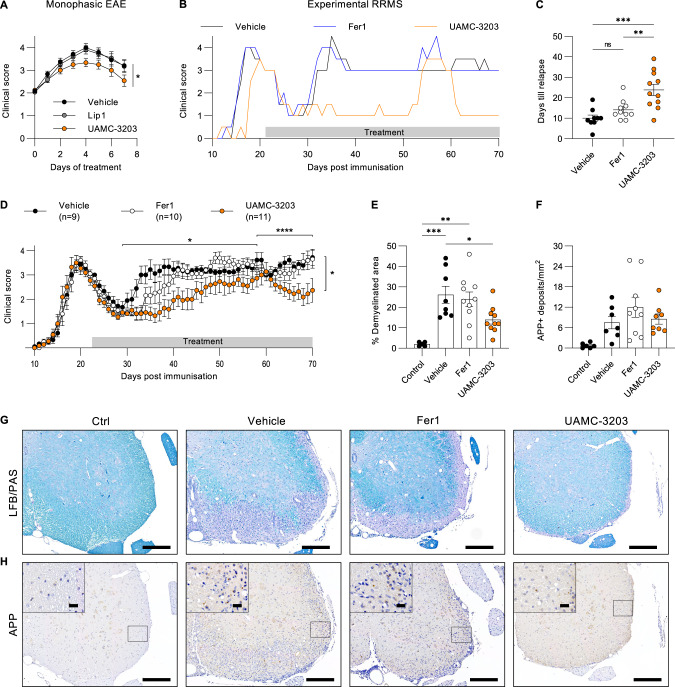
Fer1-analogue UAMC-3203 delays onset of relapse and improves early progression. **A** Clinical disease progression of monophasic EAE induced in C57Bl/6 N mice. Daily intraperitoneal treatment with vehicle (*n* = 6), Lip1 (*n* = 5) or UAMC-3203 (*n* = 6) started from score 2. **B** Representative clinical disease progression of an EAE induced Biozzi ABH mouse treated with vehicle (black), Fer1 (blue) or UAMC-3203 (orange), starting from score 3 in remission. Number of days until onset of relapse were counted per mouse (**C**) and clinical disease scores were pooled for all EAE induced Biozzi ABH mice treated with vehicle (*n* = 9), Fer1 (*n* = 10) or UAMC-3203 (*n* = 11) (**D**). Assessment of demyelination by LFB/PAS staining (**E**, **G**) and axonal damage by APP staining (**F**, **H**) after sacrifice at day 70. Scale bar, 200 µm and zoom section, 20 µm. Data is represented as mean ± SEM. Data was analyzed by one-way ANOVA with Tukey’s correction for multiple comparisons (**C**, **E**, **F**), or by repeated measures using REML analysis (**A**, **D**) (*p ≤ 0.05, **p ≤ 0.01, ***p ≤ 0.001, ****p ≤ 0.0001). Fer1 ferrostatin-1, EAE experimental autoimmune encephalomyelitis, Lip1 liproxstatin-1, LFB Luxol fast blue, PAS Periodic acid Schiff, APP amyloid-β precursor protein.

To target ferroptosis, we have previously shown the superior in vivo efficacy of our third-generation synthetic lipophilic radical trap compound UAMC-3203 in an experimental model for multi-organ failure [31]. To address the importance of ferroptosis in the classical monophasic EAE, we first challenged GPX4 transgenic mice [37]. However, no improvement in clinical disease score could be observed compared to wild-type control mice (Fig. S8A). Also, treating mice with a high vitamin E diet did not improve EAE pathology (Supplementary Fig. S8B). However, to target ferroptosis pharmacologically, we next intervened during monophasic EAE from score 2 (loss of tail tonicity) onwards by daily treatment with UAMC-3203. This revealed a significant improvement of disease compared to vehicle or treatment with liproxstatin-1 (Lip1) (Fig. 5A). Because monophasic EAE doesn’t allow studying the therapeutic effect of UAMC-3203 between relapses, we next used the experimental RRMS model in Biozzi ABH mice. Treatment was initiated during remission as soon as mice gained weight and reached a clinical disease score of 3 or lower. Interestingly, daily intraperitoneal administration of UAMC-3203 significantly lowered the overall clinical disease score during the relapse remitting disease course (Fig. 5B, D). In addition, stable body weight was observed in UAMC-3203 treated mice (Fig. S8C). Quantification of the number of days from the start of treatment till the onset of relapse showed a significant delay of relapse in UAMC-3203 treated mice compared to vehicle and Fer1 treated mice (Fig. 5C). Also, the delay of relapse upon UAMC-3203 treatment was higher in female mice compared to male littermates (Fig. S8D). Next, assessment of the demyelinated area by LFB/PAS showed significantly reduced damage in the lumbar spinal cords of UAMC-3203 treated mice compared to vehicle or Fer1 treated mice (Fig. 5E, G). The number of APP deposits, as a marker of axonal damage, did not show any difference between vehicle, Fer1, or UAMC-3203 treated groups (Fig. 5F, H). Lastly, after 2–3 relapses, around 58 days post-immunization, mice developed an early progressive disease course characterized by stable paresis or paralysis from which they do not recover anymore [38, 39]. Together, these data show that treatment with UAMC-3203 protected against the early progressive disease course compared to vehicle and Fer1-treated mice (Fig. 5B, D). In summary, these findings identify ferroptosis as a detrimental factor in MS pathology, which could be targeted. Altogether, this creates the rationale for novel treatment options to control progressive injury along with current immunosuppressive strategies.

## Discussion

Different modes of RCD have been observed in human MS (histo)pathology or suggested to contribute to MS pathology based on experimental models of monophasic EAE e.g. apoptosis [40], necroptosis [7], parthanatos [41], pyroptosis [42] and netosis [43]. The observed protection against monophasic EAE typically reflects a defect in the initiation of MS pathology (auto-immune attack), rather than therapeutic control of cell death-driven injury. Here, we identify ferroptosis, an iron-catalyzed mode of cell death, as a crucial form of RCD that plays a detrimental role in demyelination and disease progression during RRMS and progressive MS. Knowing that excessive iron is toxic to neurons and oligodendrocytes [44], it is proposed that iron overload may play a harmful role in MS pathology. In line with previous studies, we show the accumulation of iron in the rim of chronic lesions and in the vicinity of active lesion sites, while iron levels are lower in NAWM of MS patients [32, 45]. The redox-active ferrous iron, Fe^2+^, drives ferroptosis by reacting with hydrogen peroxide and the formation of highly reactive hydroxyl radicals. We refined the observation on the possible role of iron by revealing that especially Fe^2+^ is accumulating in actively demyelinating MS lesions and in the rim of chronic lesions. Furthermore, we demonstrate for the first time that in CSF of MS patients, the Fe^2+^/Fe^3+^ ratios are elevated compared to non-MS controls. The exact underlying mechanism that causes this disrupted iron homeostasis in the injured CNS is not known. It is conceivable that active disruption of the myelin sheath and death of myelin-producing oligodendrocytes, infiltrating inflammatory cells, and the release of heme (by increased expression of heme oxygenase-1 (HMOX1), data not shown) could drive disruption of iron homeostasis.

Excessive lipid peroxidation is considered the main executioner mechanism leading to ferroptotic cell death, which doesn’t occur in other types of regulated cell death [46]. Adding on previous observations related to oxidative lipid damage [47–51], we detected oxidized phosphatidylcholines in active and chronic MS lesions as well as their truncated variants in CSF. The increased levels of Fe^2+^ could participate in the decomposition of lipid hydroperoxides to produce the oxidized truncated phospholipids [52, 53]. These oxidized phosphatidylcholines contribute to ferroptosis by disturbing mitochondrial function and suppressing GPX4 activity, shown in cardiomyocytes during ischemia-reperfusion injury [54]. Determining a unique ferroptosis-specific epilipidome signature remains a major challenge, which is topic of future research. We also found an extended signature of 4-HNE modified proteins, which typically follows excessive lipid peroxidation. We found a drop in GPX4 mRNA and protein levels during the acute phase in relapsing-remitting EAE, which was previously observed in monophasic EAE as well as in biopsies of MS patients [36, 55]. Recently, other ferroptosis protective mechanisms such as FSP1 [20, 56] and GCH1 [19] have been discovered, which often compensate for GPX4 reduction. Indeed, when GPX4 is diminished during the acute phase, we observed a compensatory transcriptional increase of *Fsp1* and *Gch1* mRNA. The reduced levels of glutathione observed in MS patients might drive loss of GPX4 [18]. Alternatively, GPX4 might be degraded by the ubiquitin proteasome system due to its oxidation, as observed in experimental mouse model for Parkinson’s disease [57].

To mimic progressive RRMS in patients, we have set up a relapsing-remitting EAE in Biozzi ABH mice, which also allows therapeutic intervention. Upon EAE induction, mice develop a relapsing-remitting disease course followed by a phenotypically stable chronic progressive phase [25, 39]. We found that CD3^+^ T cells and macrophages start to infiltrate early during acute disease while their numbers decline during remission. However, B cell infiltration accumulates during disease progression. The inflammatory influx of B and T cells suggests that acute immune attacks are dominantly driven by T cell infiltration, whereas chronic disease progression is associated with B cell infiltration. Note that although B cells contribute to MS pathology in humans, it is known that this is not well reflected in experimental rodent MS models [58].

Initiation of cell death occurs as an early event and progressively increases during the disease course. This might be due to Wallerian and neuronal degeneration that is initiated as soon as axons are intersected during the acute phase of disease [34, 35]. We confirmed a ferroptosis signature in relapsing-remitting EAE mice, in agreement with our observations in active and chronic lesions of MS patients, suggesting ferroptosis as a detrimental factor in MS and relapsing-remitting EAE. Indeed, inhibition of ferroptosis using our third-generation ferrostatin-analog UAMC-3203 [59] ameliorates experimental relapsing-remitting EAE. We show that UAMC-3203 outcompetes Fer1 (and Lip1 in monophasic EAE) by lowering the overall clinical disease score, as well as increasing the time interval before relapse. This clinical improvement is likely due to a strong drop in demyelination in the injured spinal cord upon UAMC-3203 treatment. Hypothetically, this drop in demyelination might circumvent the loss of fatty acid degradation by astrocytes as a trigger for neurodegeneration [60]. Note that ferroptosis was recently also reported as mode of action in cuprizone-induced demyelination [61]. Conceptually, our findings not only suggest a role for ferroptosis in disease progression but also in relapse, which might imply an interplay between ferroptosis and the initiation of autoimmune attacks by modulating their activation [62] or differentiation. Alternatively, inhibition of lipid peroxidation might also directly affect ferroptosis in either T [63] or B cells [64]. Although we observed a strong decrease in demyelination, no drop in APP deposits was found. Because APP deposition is a very late event following injury, we assume that this APP deposition rather reflects injury of the first immune attack (day 10–25), when treatment was not started yet. Finally, to improve this therapeutic effect even further, we are currently developing novel potent ferroptosis inhibitors that cross the blood-brain barrier. Because UAMC-3203 doesn’t cross the blood-brain barrier [31], we assume it is taken up at the time of blood-brain barrier disruption during the acute relapse phase.

In conclusion, the observation of dysregulated iron homeostasis, increase in labile iron, and excessive lipid peroxidation in lesions and CSF of MS patients put forward ferroptosis as an important detrimental factor in MS disease. The potent protective effect of UAMC-3203 in an experimental RRMS model strongly suggests that inhibition of ferroptosis could be a new therapeutic strategy to treat MS by damping lipid peroxidation in the tightly packed myelin phospholipid layers and favoring appropriate neuron functioning. Along with immunosuppressive strategies [2], synthetic lipophilic radical traps that cross the blood-brain barrier could be considered as novel treatment options controlling oligodendrocyte and neuronal cell death.

## Materials and methods

### Patient specimens

Frozen CSF and paraffin-embedded brain tissue blocks were purchased at the Netherlands Brain Bank (NBB). Before sample selection, preliminary pathological staging and categorization of MS lesions was provided by the NBB via PLP/HLA staining procedures [26]. Paraffin-embedded brain blocks were chosen upon the presence of at least one active and one mixed active/inactive (chronic) lesion. In total, 12 SPMS patient donors and 10 non-demented control donors were selected. For each of the samples, post-mortem delay (PMD) was limited to ±10 h to minimize degradation and cell death. Details regarding the type of specimen, age, sex, PMD, and lesion types are listed in Table S1.

### Laboratory mice

Biozzi ABH mice were originally purchased from Envigo and bred in-house under SPF conditions in individually ventilated cages in temperature controlled (21 °C) animal facilities with 14/10 h light/dark cycles and a 55% humidity-controlled environment. C57Bl/6 N mice were ordered from Janvier Labs, and an acclimatization period of 3 weeks was included before any experiment. *Gpx4*^Tg/+^ mice were kindly provided by Q. Ran [37], in each experiment genetically modified mice were compared to wild-type littermates. Animals were provided with water and normal chow *ad libitum*. Except for animals on an adapted vitamin E diet, synthetic pellets (Sniff) were administered from the age of 4 weeks until the end of the experiment.

### Genotyping

*Gpx4*^*Tg/+*^ mice were characterized using PCR primers: 5′-CGTGGAACTGTGAGCTTTGTG-3′ and 5′- AAGGATCACAGAGCTGAGGCTG-3′, yielding a 300 bp DNA fragment upon overexpression of GPX4.

### Induction of relapsing-remitting EAE

Relapsing-remitting progressive EAE was induced as described before [39]. Briefly, 6/8-week-old male and female Biozzi ABH mice were immunized with an emulsion of spinal cord homogenate (SCH) in incomplete Freund’s adjuvant (IFA), supplemented with *Mycobacterium tuberculosis* H37RA (4 mg/ml) and *Mycobacterium butyricum* (0,5 mg/ml). Mice were subcutaneously inoculated at day 0 and day 7 and monitored daily from day 10 onwards. Clinical disease scoring was done by a 6-point scoring system: 0 no symptoms; 1 weakness of tail point; 2 loss of tail tonicity; 3 partial hind limb paralysis; 4 hind limb paralysis, 5 forelimb paralysis/moribund, and 6 spontaneous death. For kinetic characterization, mice were sacrificed during the pre-acute, acute, remitting, and relapsing phase, and age-matched littermates were sacrificed as control. No differences in clinical disease scores were observed between male and female Biozzi ABH mice upon EAE induction. Mice sacrificed during the pre-acute phase, 13–14 days post-immunization (p.i.), reflected an initial weight loss and first clinical symptoms such as weakness of the tail point. During acute disease, 16–20 days p.i., complete paralysis of hind limbs was observed. Animals recovered nearly completely around day 23–24 p.i., but still showed some residual loss of tail tonicity. A following drop in body weight and worsening of symptoms between day 30–42 p.i indicated the onset of relapse. After 2–3 relapses, around 58 days p.i., mice developed an early chronic progressive disease course characterized by stable paresis or paralysis from which they did not recover anymore.

### Induction of monophasic EAE

To induce monophasic EAE, 8/15-week-old male littermate mice were immunized by subcutaneous injection with an emulsion of 200 µg myelin oligodendrocyte glycoprotein (MOG_35–55_) peptide in 100 µl sterile phosphate buffered solution (PBS) and an equal volume of complete Freund’s adjuvant (CFA), supplemented with *M. tuberculosis* H37RA. 48 h p.i. mice received an additional intraperitoneal injection of 50 ng pertussis toxin (Sigma-Aldrich) in 200 µl sterile PBS. Mice were monitored daily, and clinical scoring was performed as mentioned before with a 6-point scaling system.

### Drug administration

To investigate therapeutic intervention with ferroptosis inhibitors in relapsing-remitting EAE, immunized Biozzi ABH mice received treatment during remission as soon as mice gained weight and reached a clinical score of 3 or lower. In contrast, intervention during monophasic EAE was initiated during the acute attack, from score 2 (loss of tail tonicity) onwards. Ferroptosis inhibition was performed by daily treatment of Fer1 or Lip1 and UAMC-3203, equimolar amounts of the compounds were dissolved in 2% dimethyl sulfoxide (DMSO, Sigma) in 0.9% sterile NaCl. A total concentration of 2 mM was injected intraperitoneally (IP) in a volume of 200 µl/20 g body weight, equivalent to 5.2 mg/kg Fer1, 6.8 mg/kg Lip1, and 12.35 mg/kg UAMC-3203. Vehicle-treated mice received daily IP injections of 200 µl/20 g 2% DMSO in 0.9% sterile NaCl. The site of injection was alternated daily to avoid scar formation. To avoid diagnostic bias, mice were scored blindly.

### Histological analysis

Mice were transcardially perfused via the left ventricle with PBS containing 5 IU/ml heparin, followed by perfusion with 4% paraformaldehyde. Spinal cords were isolated, dehydrated, and embedded in paraffin blocks. Human and/or mouse paraffin sections of 5 µm were stained with Luxol fast blue (Solvent Blue 38, practical grade, Sigma Genosys) followed by Periodic acid Schiff (PAS) staining (Sigma) for assessment of demyelination or immunohistochemically stained with antibodies against amyloid precursor protein (clone 22C11; Merck), B220 (clone RA3-6B2; ThermoFischer Scientific), CD3 (clone CD3-12; Bio-Rad), CD45 (ab10558; Abcam), oxidized phosphatidylcholine (E06 mAB-biotinylated, 330002 S; Avanti Polar Lipids), 4-HNE (HNEJ-1 antibody, staining was kindly performed by Shinya Toyokuni [29]), GPX4 (ab125066; Abcam) and Mac-3 (clone M3/84, Becton Dickinson Pharmingen). Subsequently, sections were stained with hematoxylin as counterstaining. Cell death was visualized by terminal deoxynucleotidyl transferase dUTP nick end labeling (TUNEL) according to the manufacturer’s instructions (In situ cell death detection kit, TMR red; Roche Diagnostics). Visualization of total non-heme iron and ferrous iron was performed as described formerly by Hametner et al. [32].

Bright-field and immunofluorescent imaging was performed using an Axio Scan.Z1 (Zeiss, Germany). Only lumbar spinal cord sections [35] were quantified for analysis by running scripts on QuPath Software (v0.3.0), with the kind help of VIB Bioimaging Core (Ghent Belgium).

### Quantitative real-time PCR on mouse tissue

Total RNA was isolated from complete spinal cord tissue using TRIzol reagent (Invitrogen) and an Aurum Total RNA Isolation Mini Kit (Bio-Rad), according to the manufacturer’s instructions. Synthesis of cDNA was performed using a SensiFAST cDNA Synthesis kit (Bioline), according to the manufacturer’s instructions. A total of 10 ng cDNA was used for quantitative PCR in a total volume of 10 µl with LightCycler 480 SYBR Green I Master Mix (Roche) and specific primers, on a LightCycler 480 (Roche). Real-time PCR reactions were performed in triplicates. The following mouse-specific primers were used: CHAC1 forward, 5′-TACGGCTCCCTAGTGTGGAA-3′, CHAC1 reverse, 5′-CTCGGCCAGGCATCTTGT-3′; FSP1 forward, 5′-CATGGTGATTGTGTGCAATGG-3′, FSP1 reverse, 5′-TGGTCAGTCTCTGGCTTGTAAG-3′; GCH1 forward, 5′-ACTTCACCAAGGGATACCAGG-3′, GCH1 reverse, 5′-CTTGCTTGTTAGGAAGATAGCCA-3′; GPX4 forward, 5′-GCAAGCCATACTCGGCCTC-3′, GPX4 reverse, 5′-GCACACGAAACCCCTGTACTT-3′; HMOX-1 forward, 5′-TAGCCCACTCCCTGTGTTTCCTTT-3′ HMOX-1 reverse, 5′-TGCTGGTTTCAAAGTTCAGGCCAC-3′; HSPB1 forward, 5′-ATCACTGGCAAGCACGAAGA-3′, HSPB1 reverse, 5′-ATCACTGGCAAGCACGAAGA-3′; PTGS2 forward, 5′-GGGCCATGGAGTGGACTTAAA-3′, PTGS2 reverse, 5′-GGGATACACCTCTCCACCAAT-3′ and TFR1 forward, 5-′AAACTGGCTGAAACGGAGGAGACA-3′, TFR1 reverse, 5′- GCTGCTTGATGGTGTCAGCAAACT-3′.

### CE-ICP-DRC-MS

Iron redox species Fe^2+^, Fe^3+,^ and ferritin iron load were quantified by capillary electrophoresis- inductively coupled plasma mass spectrometry with dynamic reaction cell (CE-ICP-DRC-MS) [33, 65, 66]. A *"PrinCe 706”* capillary electrophoresis (CE) system from PrinCe Technologies B.V. (Emmen, The Netherlands) was used for species separation. Temperature settings for the sample/buffer tray and the capillary were 20^°^C by integrated air temperature control.

The capillary (CS-Chromatographie Service GmbH, Langerwehe, Germany) for separation and hyphenation to ICP-MS as the element selective detector was uncoated with dimension 85 cm × 50 µm ID.

The CE-ICP-MS interface from [33] was installed at ICP-MS sample entrance providing electrical connection for the CE separation circuit and reduction of the undesired suction. Diluted hydrochloric acid (5 mM) was used as the auxiliary sheath liquid. A *“NexIon 300-D”* ICP-DRC-MS system from Perkin Elmer (Rodgau-Jügesheim, Germany) was operated on-line as the element selective detector for iron electropherograms. The isotopes ^56^Fe and ^57^Fe were measured in dynamic reaction cell (DRC) mode with ammonia as DRC gas (0.58 ml NH_3_/min). DRC rejection parameter was set to 0.58, and the dwell time to 50 ms. The RF power was 1250 W, and the plasma gas was 16 L Ar/min. The nebulizer gas at the CE-ICP-DRC-MS interface was optimized and finally set to 0.98 L Ar/min. Electropherograms were exported from ICP-MS software (Syngistix, Perkin Elmer) to Peakfit^TM^ (Systat, Inpixon GmbH Office, Düsseldorf, Germany) for peak area integration and quantification.

For iron recovery checks we analyzed total iron with ICP-sectorfield mass spectrometry (ICP-sf-MS) in medium resolution mode at ^56^Fe and ^57^Fe iron isotopes. The experimental settings for ICP-sf-MS (“*ELEMENT 2”*, Thermo Scientific, Bremen, Germany) were: Radio frequency power: 1260 W, plasma gas flow: 16 L Ar/min auxiliary gas flow: 0.85 L Ar/min, nebulizer gas flow: 1.085 L Ar/min, daily optimized, dwell time 300 ms. The sum of quantified iron species from CE-ICP-DRC-MS was compared to total iron (set 100%) and ranged between 92–110%.

### Colorimetric MDA assay

As described previously [67], an estimation of malondialdehyde (MDA) levels was made via colorimetric measurement of 1-methyl-2-phenylindole reactive species. Briefly, 100 µl of CSF was added to 325 µl reagent solution containing 10 mM 1-methyl-2-phenylindole (sc-253936; Santa Cruz) dissolved in acetonitrile/methanol (3:1). Colorimetric reaction was initiated by adding 75 µl fresh hydrochloric acid (37%) and incubation at 70 °C for 45 min. Afterward, samples were centrifuged at 4 °C for 10 min at 15000 *g* to terminate the reaction. The supernatant was collected and measured at 595 nm absorbance. A standard of 1,1,3,3-tetramethoxypropane (108383; Sigma-Aldrich) was used to evaluate the MDA concentration.

### LC/ESI-MS analysis of phosphatidylethanolamine (PE) and phosphatidylcholine (PC)

Total lipids were extracted from CSF by using Bligh and Dyer procedure [68]. LC/ESI-MS analyses of PE and its oxygenated species were performed on a Thermo HPLC system coupled to a Thermo Scientific™ Orbitrap Fusion™ Lumos™ Tribrid™ mass spectrometer. Phospholipids were separated on a normal phase column (Luna 3 μm Silica (2) 100 A, 150 × 1.0 mm, (Phenomenex)) at a flow rate of 0.065 ml/min. The column was maintained at 35 °C. The analysis was performed using gradient solvents as previously described [69]. PE was analyzed in negative ion mode using the following parameters: capillary voltage, 3500; sheath, aux, and sweep gases (35, 17, 0, respectively); ion transfer tube temperature, 300 °C; orbitrap resolution, 120,000; scan range 400–1800 m/z; Rf lens, 40; injection time, 100 ms; intensity threshold set at 4e^2^. For data-dependent MS^2^, an isolation window of 1.2 m/z was used. Collision energy (HCD) was static at 24 with an orbitrap resolution of 15,000 with an injection time of 22 ms. Compound Discoverer^TM^ software package (ThermoFisher Scientific) with an in-house generated analysis workflow and oxidized phospholipid database was used to evaluate LC/MS data. Peaks with a signal/noise ratio of >3 were identified and searched against the database of oxidized phospholipids. PE signals were further filtered by retention time and values for *m/z* were matched within 5 ppm to identify the PE species. For many species, the identification of PE species was confirmed by MS^2^ analysis. Exact mass and retention time were used for the identification of PE and PC truncated species. PC truncated species were detected as adducts with formic acid. Commercially available 1-stearoyl-2-15(S)-HpETE-sn-glycero-3-phosphoethanolamine, 1-stearoyl-2-15(S)-HETE-sn-glycero-3-phospho-ethanolamine (Cayman Chemicals), 1-stearoyl-2-arachidonoyl-sn-glycero-3-phosphoethano-lamine, 1,2-dioleoyl-sn-glycero-3-phospho-ethanolamine, 1-palmitoyl-2-(5′-oxo-valeroyl)-sn-glycero-3-phosphocholine, 1-palmitoyl-2-(9′-oxo-nonanoyl)-sn-glycero-3-phosphocholine (Avanti Polar Lipids) were used as reference standards. Deuterated PE and PC: 1-hexadecanoyl(d31)-2-(9Z-octadecenoyl)-sn-glycero-3-phosphoethanolamine, 1-palmitoyl-d31-2-oleoyl-sn-glycero-3-phosphocholine (Avanti Polar Lipids) was used as internal standard. The internal standard was added directly to the MS sample to a final concentration of 1 µM.

### Statistics

Sample sizes were determined using G power 3.9.1.7 software and statistical analysis was performed using Graphpad Prism 9.4.0. Outliers were identified and results are expressed as mean ± SEM. Statistical analysis between two groups was assessed using a nonparametric Mann–Whitney U-test or two-tailed unpaired *t*-test, depending on the outcome of the normality by the Shapiro-Wilk test. Assumption of variance was validated by an F-test. Quantitative results from pre-acute, acute, remission and relapse samples were checked for normality and analyzed using one-way ANOVA with Tukey’s correction for multiple comparisons, or with the Kruskal-Wallis test followed by Dunn’s multiple comparison test. Clinical scores were analyzed as repeated measurements using a method of residual maximum likelihood (REML), as implemented in Genstat version 22. Briefly, a linear mixed model (random terms underlined) of the form: Score=constant+treatment+time+treatment×time+subject×time was fitted to the clinical score data. The term subject×time represents the residual error term with dependent errors because the repeated measurements are taken in the same individual, causing correlations among observations. The autoregressive correlation structure of order 1 (AUTO1), allowing serial correlation within subjects, was selected as the best model fit based on the Akaike Information Coefficient. Additional options selected to get a best-fitting model included 1) times of measurement were set as equally spaced, and 2) allowance of unequal variances across time. The significance of the fixed main and interaction terms in the model were assessed using a Wald test as implemented in Genstat version 22. Pairwise comparisons between treatments across the two-time series D29-D58 and D58-D70 were assessed by a Wald test. Additionally, one- or two-way ANOVA and Tukey’s correction for multiple comparisons was applied to statistically quantify the number of days till the onset of relapse.

## Supplementary information

Reproducibility checklist

Supplementary Information

## Data Availability

All data generated or analysed during this study are included in this published article [and its supplementary information files].

## References

[1] Walton C, King R, Rechtman L, Kaye W, Leray E, Marrie RA (2020). Rising prevalence of multiple sclerosis worldwide: Insights from the Atlas of MS, third edition. Mult Scler.

[2] McGinley MP, Goldschmidt CH, Rae-Grant AD (2021). Diagnosis and Treatment of Multiple Sclerosis: A Review. JAMA..

[3] Guire C, Volckaert T, Wolke U, Sze M, Rycke RD, Waisman A (2010). Oligodendrocyte-Specific FADD Deletion Protects Mice from Autoimmune-Mediated Demyelination. J Immunol.

[4] McKenzie BA, Mamik MK, Saito LB, Boghozian R, Monaco MC, Major EO (2018). Caspase-1 inhibition prevents glial inflammasome activation and pyroptosis in models of multiple sclerosis. Proc Natl Acad Sci..

[5] Humphries F, Shmuel-Galia L, Ketelut-Carneiro N, Li S, Wang B, Nemmara VV (2020). Succination inactivates gasdermin D and blocks pyroptosis. Science..

[6] Coll RC, Robertson AA, Chae JJ, Higgins SC, Munoz-Planillo R, Inserra MC (2015). A small-molecule inhibitor of the NLRP3 inflammasome for the treatment of inflammatory diseases. Nat Med.

[7] Ofengeim D, Ito Y, Najafov A, Zhang Y, Shan B, DeWitt JP (2015). Activation of necroptosis in multiple sclerosis. Cell Rep.

[8] Kennedy PGE, George W, Yu X (2022). The Possible Role of Neural Cell Apoptosis in Multiple Sclerosis. Int J Mol Sci.

[9] Mahad DH, Trapp BD, Lassmann H (2015). Pathological mechanisms in progressive multiple sclerosis. Lancet Neurol.

[10] Ward RJ, Zucca FA, Duyn JH, Crichton RR, Zecca L (2014). The role of iron in brain ageing and neurodegenerative disorders. Lancet Neurol.

[11] Yang WS, Stockwell BR (2016). Ferroptosis: Death by Lipid Peroxidation. Trends Cell Biol.

[12] Dixon SJ, Lemberg KM, Lamprecht MR, Skouta R, Zaitsev EM, Gleason CE (2012). Ferroptosis: an iron-dependent form of nonapoptotic cell death. Cell..

[13] Riegman M, Sagie L, Galed C, Levin T, Steinberg N, Dixon SJ (2020). Ferroptosis occurs through an osmotic mechanism and propagates independently of cell rupture. Nat Cell Biol.

[14] Kagan VE, Mao G, Qu F, Angeli JP, Doll S, Croix CS (2017). Oxidized arachidonic and adrenic PEs navigate cells to ferroptosis. Nat Chem Biol.

[15] Aggarwal S, Yurlova L, Simons M (2011). Central nervous system myelin: structure, synthesis and assembly. Trends Cell Biol.

[16] O’Brien JS (1965). Stability of the Myelin Membrane. Science.

[17] Yang WS, SriRamaratnam R, Welsch ME, Shimada K, Skouta R, Viswanathan VS (2014). Regulation of ferroptotic cancer cell death by GPX4. Cell..

[18] Carvalho AN, Lim JL, Nijland PG, Witte ME, Van Horssen J (2014). Glutathione in multiple sclerosis: more than just an antioxidant?. Mult Scler.

[19] Kraft VAN, Bezjian CT, Pfeiffer S, Ringelstetter L, Müller C, Zandkarimi F (2020). GTP Cyclohydrolase 1/Tetrahydrobiopterin Counteract Ferroptosis through Lipid Remodeling. ACS Cent Sci.

[20] Doll S, Freitas FP, Shah R, Aldrovandi M, da Silva MC, Ingold I (2019). FSP1 is a glutathione-independent ferroptosis suppressor. Nature..

[21] Mao C, Liu X, Zhang Y, Lei G, Yan Y, Lee H (2021). DHODH-mediated ferroptosis defence is a targetable vulnerability in cancer. Nature..

[22] Skouta R, Dixon SJ, Wang J, Dunn DE, Orman M, Shimada K (2014). Ferrostatins inhibit oxidative lipid damage and cell death in diverse disease models. J Am Chem Soc.

[23] Zilka O, Shah R, Li B, Friedmann Angeli JP, Griesser M, Conrad M (2017). On the Mechanism of Cytoprotection by Ferrostatin-1 and Liproxstatin-1 and the Role of Lipid Peroxidation in Ferroptotic Cell Death. ACS Cent Sci.

[24] Yang WS, Stockwell BR (2008). Synthetic lethal screening identifies compounds activating iron-dependent, nonapoptotic cell death in oncogenic-RAS-harboring cancer cells. Chem Biol.

[25] Baker D, O’Neill JK, Gschmeissner SE, Wilcox CE, Butter C, Turk JL (1990). Induction of chronic relapsing experimental allergic encephalomyelitis in Biozzi mice. J Neuroimmunol.

[26] Luchetti S, Fransen NL, van Eden CG, Ramaglia V, Mason M, Huitinga I (2018). Progressive multiple sclerosis patients show substantial lesion activity that correlates with clinical disease severity and sex: a retrospective autopsy cohort analysis. Acta Neuropathol.

[27] Prineas JW, Parratt JDE (2021). Multiple Sclerosis: Microglia, Monocytes, and Macrophage-Mediated Demyelination. J Neuropathol Exp Neurol.

[28] Palinski W, Horkko S, Miller E, Steinbrecher UP, Powell HC, Curtiss LK (1996). Cloning of monoclonal autoantibodies to epitopes of oxidized lipoproteins from apolipoprotein E-deficient mice. Demonstration of epitopes of oxidized low density lipoprotein in human plasma. J Clin Invest.

[29] Toyokuni S, Miyake N, Hiai H, Hagiwara M, Kawakishi S, Osawa T (1995). The monoclonal antibody specific for the 4-hydroxy-2-nonenal histidine adduct. Febs Lett.

[30] Hassannia B, Wiernicki B, Ingold I, Qu F, Van Herck S, Tyurina YY (2018). Nano-targeted induction of dual ferroptotic mechanisms eradicates high-risk neuroblastoma. J Clin Invest.

[31] Van Coillie S, Van San E, Goetschalckx I, Wiernicki B, Mukhopadhyay B, Tonnus W (2022). Targeting ferroptosis protects against experimental (multi)organ dysfunction and death. Nat Commun.

[32] Hametner S, Wimmer I, Haider L, Pfeifenbring S, Bruck W, Lassmann H (2013). Iron and neurodegeneration in the multiple sclerosis brain. Ann Neurol.

[33] Michalke B, Willkommen D, Venkataramani V (2019). Iron Redox Speciation Analysis Using Capillary Electrophoresis Coupled to Inductively Coupled Plasma Mass Spectrometry (CE-ICP-MS). Front Chem.

[34] Lassmann H, Bradl M (2017). Multiple sclerosis: experimental models and reality. Acta Neuropathol.

[35] Jackson SJ, Lee J, Nikodemova M, Fabry Z, Duncan ID (2009). Quantification of myelin and axon pathology during relapsing progressive experimental autoimmune encephalomyelitis in the Biozzi ABH mouse. J Neuropathol Exp Neurol.

[36] Hu CL, Nydes M, Shanley KL, Pantoja IE, Howard TA, Bizzozero OA. Reduced expression of the ferroptosis inhibitor GPx4 in multiple sclerosis and experimental autoimmune encephalomyelitis. J Neurochem. 2018;148:426–39 10.1111/jnc.14604 PMC6347488 30289974

[37] Ran Q, Liang H, Gu M, Qi W, Walter CA, Roberts LJ (2004). Transgenic mice overexpressing glutathione peroxidase 4 are protected against oxidative stress-induced apoptosis. J Biol Chem.

[38] Hampton DW, Serio A, Pryce G, Al-Izki S, Franklin RJ, Giovannoni G (2013). Neurodegeneration progresses despite complete elimination of clinical relapses in a mouse model of multiple sclerosis. Acta Neuropathol Commun.

[39] Al-Izki S, Pryce G, O’Neill JK, Butter C, Giovannoni G, Amor S (2012). Practical guide to the induction of relapsing progressive experimental autoimmune encephalomyelitis in the Biozzi ABH mouse. Mult Scler Relat Disord.

[40] Macchi B, Marino-Merlo F, Nocentini U, Pisani V, Cuzzocrea S, Grelli S (2015). Role of inflammation and apoptosis in multiple sclerosis: Comparative analysis between the periphery and the central nervous system. J Neuroimmunol.

[41] Farez MF, Quintana FJ, Gandhi R, Izquierdo G, Lucas M, Weiner HL (2009). Toll-like receptor 2 and poly(ADP-ribose) polymerase 1 promote central nervous system neuroinflammation in progressive EAE. Nat Immunol.

[42] Voet S, Mc Guire C, Hagemeyer N, Martens A, Schroeder A, Wieghofer P (2018). A20 critically controls microglia activation and inhibits inflammasome-dependent neuroinflammation. Nat Commun.

[43] Zhang H, Ray A, Miller NM, Hartwig D, Pritchard KA, Dittel BN (2016). Inhibition of myeloperoxidase at the peak of experimental autoimmune encephalomyelitis restores blood-brain barrier integrity and ameliorates disease severity. J Neurochem.

[44] Kress GJ, Dineley KE, Reynolds IJ (2002). The relationship between intracellular free iron and cell injury in cultured neurons, astrocytes, and oligodendrocytes. J Neurosci.

[45] Stephenson E, Nathoo N, Mahjoub Y, Dunn JF, Yong VW (2014). Iron in multiple sclerosis: roles in neurodegeneration and repair. Nat Rev Neurol.

[46] Wiernicki B, Dubois H, Tyurina YY, Hassannia B, Bayir H, Kagan VE (2020). Excessive phospholipid peroxidation distinguishes ferroptosis from other cell death modes including pyroptosis. Cell Death Dis.

[47] Schuh C, Wimmer I, Hametner S, Haider L, Dam A-MM, Liblau RS (2014). Oxidative tissue injury in multiple sclerosis is only partly reflected in experimental disease models. Acta Neuropathologica.

[48] Dong Y, D’Mello C, Pinsky W, Lozinski BM, Kaushik DK, Ghorbani S (2021). Oxidized phosphatidylcholines found in multiple sclerosis lesions mediate neurodegeneration and are neutralized by microglia. Nat Neurosci.

[49] Toshniwal PK, Zarling EJ (1992). Evidence for increased lipid peroxidation in multiple sclerosis. Neurochem Res.

[50] Gonzalo H, Brieva L, Tatzber F, Jove M, Cacabelos D, Cassanye A (2012). Lipidome analysis in multiple sclerosis reveals protein lipoxidative damage as a potential pathogenic mechanism. J Neurochem.

[51] Koch M, Mostert J, Arutjunyan AV, Stepanov M, Teelken A, Heersema D (2007). Plasma lipid peroxidation and progression of disability in multiple sclerosis. Eur J Neurol.

[52] Bayir H, Anthonymuthu TS, Tyurina YY, Patel SJ, Amoscato AA, Lamade AM (2020). Achieving Life through Death: Redox Biology of Lipid Peroxidation in Ferroptosis. Cell Chem Biol.

[53] Stoyanovsky DA, Tyurina YY, Shrivastava I, Bahar I, Tyurin VA, Protchenko O (2019). Iron catalysis of lipid peroxidation in ferroptosis: Regulated enzymatic or random free radical reaction?. Free Radic Biol Med.

[54] Stamenkovic A, O’Hara KA, Nelson DC, Maddaford TG, Edel AL, Maddaford G (2021). Oxidized phosphatidylcholines trigger ferroptosis in cardiomyocytes during ischemia-reperfusion injury. Am J Physiol Heart Circ Physiol.

[55] Li X, Chu Y, Ma R, Dou M, Li S, Song Y (2022). Ferroptosis as a mechanism of oligodendrocyte loss and demyelination in experimental autoimmune encephalomyelitis. J Neuroimmunol.

[56] Bersuker K, Hendricks JM, Li Z, Magtanong L, Ford B, Tang PH (2019). The CoQ oxidoreductase FSP1 acts parallel to GPX4 to inhibit ferroptosis. Nature..

[57] Sun J, Lin XM, Lu DH, Wang M, Li K, Li SR, et al. Midbrain dopamine oxidation links ubiquitination of glutathione peroxidase 4 to ferroptosis of dopaminergic neurons. J Clin Invest. 2023;133:e165228. 10.1172/JCI165228 PMC10178840 37183824

[58] Sefia E, Pryce G, Meier UC, Giovannoni G, Baker D (2017). Depletion of CD20 B cells fails to inhibit relapsing mouse experimental autoimmune encephalomyelitis. Mult Scler Relat Disord.

[59] Devisscher L, Van Coillie S, Hofmans S, Van Rompaey D, Goossens K, Meul E (2018). Discovery of Novel, Drug-Like Ferroptosis Inhibitors with in Vivo Efficacy. J Med Chem.

[60] Mi Y, Qi G, Vitali F, Shang Y, Raikes AC, Wang T (2023). Loss of fatty acid degradation by astrocytic mitochondria triggers neuroinflammation and neurodegeneration. Nat Metab.

[61] Jhelum P, Santos-Nogueira E, Teo W, Haumont A, Lenoel I, Stys PK (2020). Ferroptosis Mediates Cuprizone-Induced Loss of Oligodendrocytes and Demyelination. J Neurosci.

[62] Luoqian J, Yang W, Ding X, Tuo QZ, Xiang Z, Zheng Z (2022). Ferroptosis promotes T-cell activation-induced neurodegeneration in multiple sclerosis. Cell Mol Immunol.

[63] Wang Y, Tian Q, Hao Y, Yao W, Lu J, Chen C (2022). The kinase complex mTORC2 promotes the longevity of virus-specific memory CD4(+) T cells by preventing ferroptosis. Nat Immunol.

[64] Muri J, Thut H, Bornkamm GW, Kopf M (2019). B1 and Marginal Zone B Cells but Not Follicular B2 Cells Require Gpx4 to Prevent Lipid Peroxidation and Ferroptosis. Cell Rep.

[65] Michalke B, Willkommen D, Venkataramani V. Setup of Capillary Electrophoresis-Inductively Coupled Plasma Mass Spectrometry (CE-ICP-MS) for Quantification of Iron Redox Species (Fe(II), Fe(III)). J Vis Exp. 2020;4:e61055. 10.3791/61055 32421003

[66] Bernhard Michalke VV, inventorEx vivo Analytical Method; International PCT Application at the European Patent Office with the file number: PCT/EP2022/0738482022.

[67] Gérard-Monnier D, Erdelmeier I, Régnard K, Moze-Henry N, Yadan J-C, Chaudière J (1998). Reactions of 1-Methyl-2-phenylindole with Malondialdehyde and 4-Hydroxyalkenals. Analytical Applications to a Colorimetric Assay of Lipid Peroxidation. Chem Res Toxicol.

[68] Bligh EG, Dyer WJ (1959). A rapid method of total lipid extraction and purification. Can J Biochem Physiol.

[69] Sun W-Y, Tyurin VA, Mikulska-Ruminska K, Shrivastava IH, Anthonymuthu TS, Zhai Y-J (2021). Phospholipase iPLA2β averts ferroptosis by eliminating a redox lipid death signal. Nat Chem Biol.

