# Supplementary material for: Ferroptosis contributes to multiple sclerosis and its pharmacological targeting suppresses experimental disease progression
Source: Cell Death Differ. 2023 Aug 4;30(9):2092–103. doi: 10.1038/s41418-023-01195-0 (PMC10482919; doi:10.1038/s41418-023-01195-0)
Supplement: Supplementary file 2 — Supplementary Information [file 41418_2023_1195_MOESM2_ESM.pdf]

Supplementary Information

Ferroptosis contributes to multiple sclerosis and its pharmacological targeting suppresses experimental disease progression

Emily Van San[1,2,3], Angela C. Debruyne[4], Geraldine Veeckmans[3], Yulia Y. Tyurina[5], Vladimir A. Tyurin[5], Hao Zheng[6], Sze Men Choi[1,2], Koen Augustyns[7], Geert van Loo[1,2], Bernhard Michalke[8], Vivek Venkataramani[9], Shinya Toyokuni[6,10], Hülya Bayir[5,11], Peter Vandenabeele[1,2,12], Behrouz Hassannia[1,2,3], Tom Vanden Berghe[1,2,3]*

[1]Department of Biomedical Molecular Biology, Ghent university, Ghent, Belgium
[2]VIB-UGent Center for Inflammation Research; Ghent, Belgium
[3]Department of Biomedical Sciences, University of Antwerp, Antwerp, Belgium
[4]Department of Human Structure and Repair, Ghent University, Ghent, Belgium
[5]Department of Environmental Health and Occupational Health, University of Pittsburgh, Pittsburgh, USA
[6]Department of Pathology and Biological Responses, Nagoya University Graduate School of Medicine, Nagoya, Japan
[7]Department of Pharmaceutical Sciences, University of Antwerp, Antwerp, Belgium
[8]Research Unit Analytical BioGeoChemistry, Helmholtz Zentrum München, Munich, Germany
[9]Department of Pathology, University Medical Center, Goettingen, Germany
[10]Center for Low-temperature Plasma Sciences, Nagoya University, Furo-cho, Chikusa, Nagoya, Japan
[11]Children's Neuroscience Institute, UPMC Children's Hospital of Pittsburgh, Pittsburgh, USA
[12]Methusalem program; Ghent University, Ghent, Belgium
*Correspondence: Tom Vanden Berghe, Universiteitsplein 1, 2610 Antwerp, Belgium, Tel. +3232659250, Tom.VandenBerghe@uantwerp.be

Table S1. Patient and tissue information

Donor	NBB donor index	Paraffin-embedded lesions	Paraffin-embedded no lesions	CSF	Sex	Age	PMD	Type of MS	Identified lesions in affected tissue by NBB
Multiple sclerosis									
1	2006-017			X	F	44	10:15	SPMS	-
2	2007-010	X			M	47	7:15	SPMS	Reactive, active, chronic
3	2007-085	X			F	66	6:00	SPMS	Reactive, active, chronic, inactive
4	2010-024			X	M	44	10:15	SPMS	-
5	2010-034			X	F	57	8:40	SPMS	-
6	2010-117	X		X	F	60	10:40	SPMS	Reactive, active, chronic
7	2016-104	X		X	F	49	8:30	SPMS	Reactive, active, chronic, inactive
8	2016-113			X	F	61	10:55	SPMS	-
9	2017-001		X		F	39	8:30	SPMS	Reactive, active, chronic
10	2017-089		X		M	78	8:50	SPMS	-
11	2018-115		X		M	56	6:15	SPMS	-
12	2019-014		X		M	65	7:15	SPMS	-
Non-demented control									
Donor	NBB donor index	Paraffin-embedded		CSF	Sex	Age	PMD	Type of MS	Identified lesions in affected tissue by NBB
13	1994-057	X			F	81	7:15	-	-
14	1995-054			X	F	72	9:10	-	-
15	1996-019	X			F	86	5:15	-	-
16	1997-116	X			M	80	6:56	-	-
17	1998-049			X	M	87	7:25	-	-
18	1998-089			X	F	90	7:15	-	-
19	2000-015			X	M	78	5:35	-	-
20	2000-032	X			F	78	6:30	-	-
21	2006-049			X	F	84	4:45	-	-
22	2009-005			X	M	82	5:10	-	-

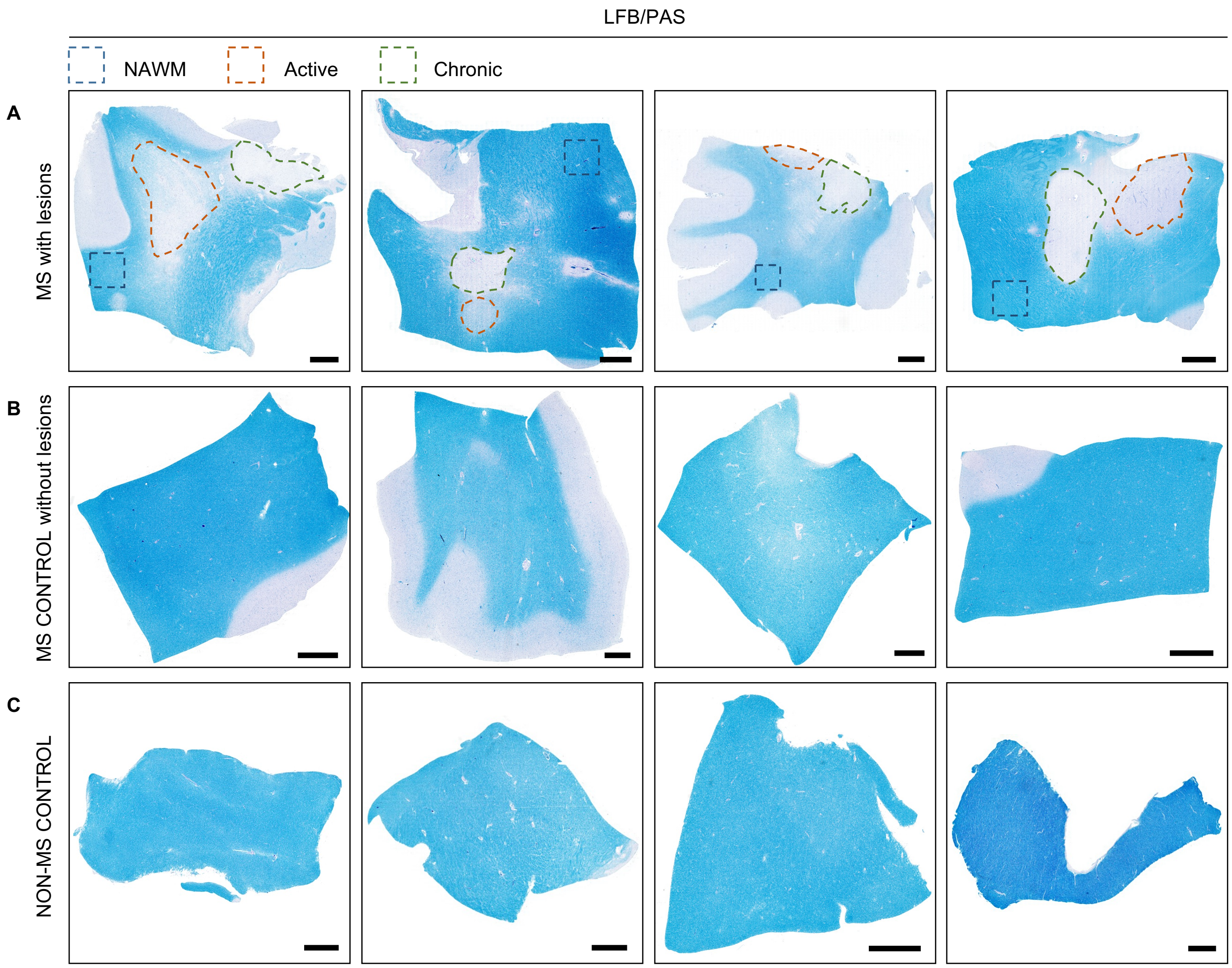

Figure S1. Paraffin-embedded sections from (**A**) lesion-containing MS, (**B**) non-lesion containing MS and (**C**) non-demented control brain tissue were stained with Luxol fast blue combined with periodic acid-Schiff (LFB/PAS). Normal appearing white matter (NAWM), active and chronic regions of interest are demarcated by blue, orange and green dotted line, respectively. n=4/group. Scale bars, 2 mm.

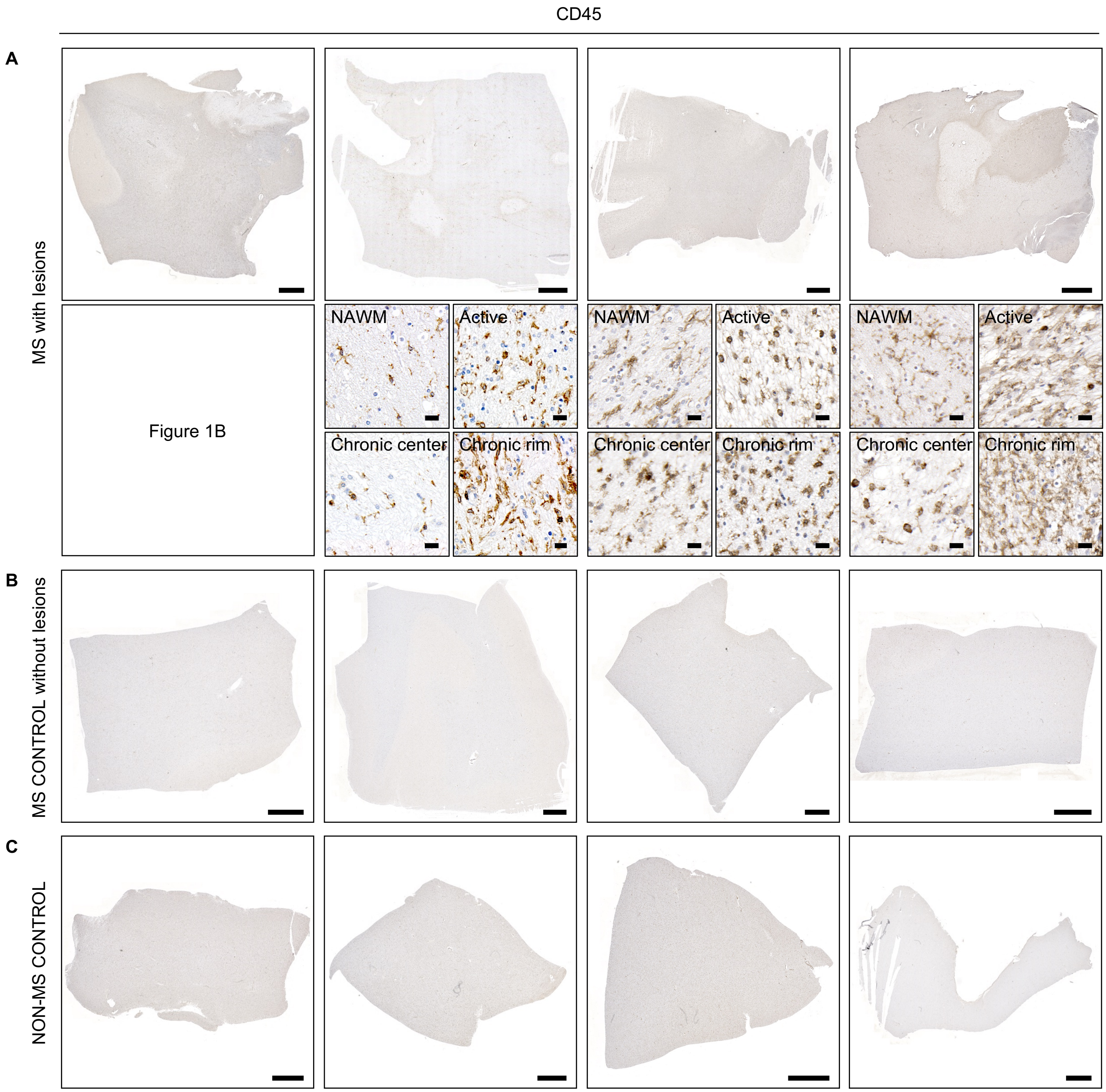

Figure S2. Paraffin-embedded sections from (**A**) lesion-containing MS, (**B**) non-lesion containing MS and (**C**) non-demented control brain tissue were stained for $CD45^{+}$ myeloid cells and microglia. n=4/group. Scale bars, 2 mm and 20 µm (zoom). Normal appearing white matter (NAWM), active and chronic regions of interest are indicated in Fig. S1 by blue, orange and green dotted line, respectively.

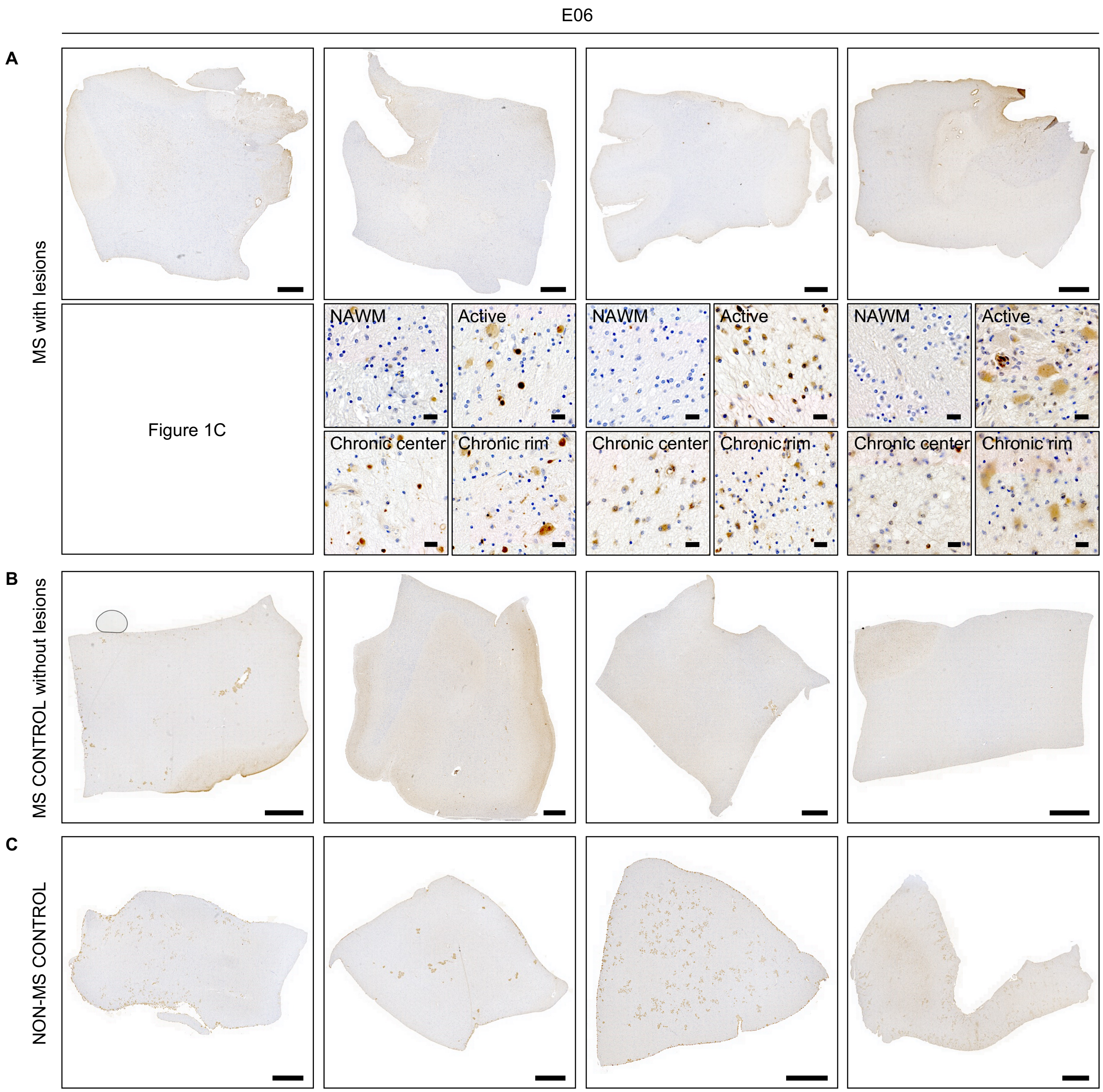

Figure S3. Paraffin-embedded sections from (**A**) lesion-containing MS, (**B**) non-lesion containing MS and (**C**) non-demented control brain tissue were stained for oxidized phosphatidylcholines (E06). n=4/group. Scale bars, 2 mm and 20 µm (zoom). Normal appearing white matter (NAWM), active and chronic regions of interest are indicated in Fig. S1 by blue, orange and green dotted line, respectively.

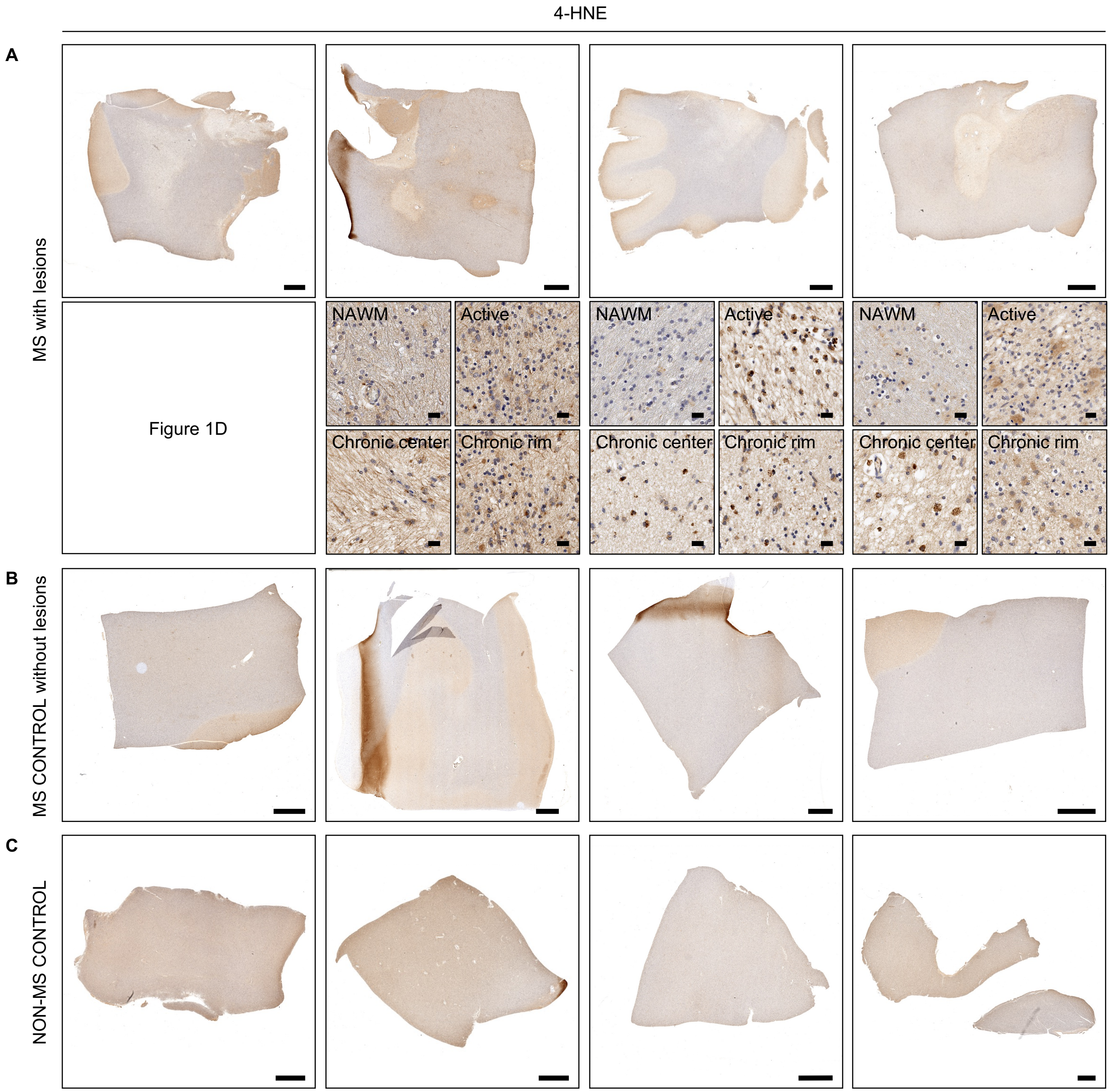

Figure S4. Paraffin-embedded sections from (**A**) lesion-containing MS, (**B**) non-lesion containing MS and (**C**) non-demented control brain tissue were stained for 4-hydroxy-2-nonenal (4-HNE). n=4/group. Scale bars, 2 mm and 20 µm (zoom). Normal appearing white matter (NAWM), active and chronic regions of interest are indicated in Fig. S1 by blue, orange and green dotted line, respectively.

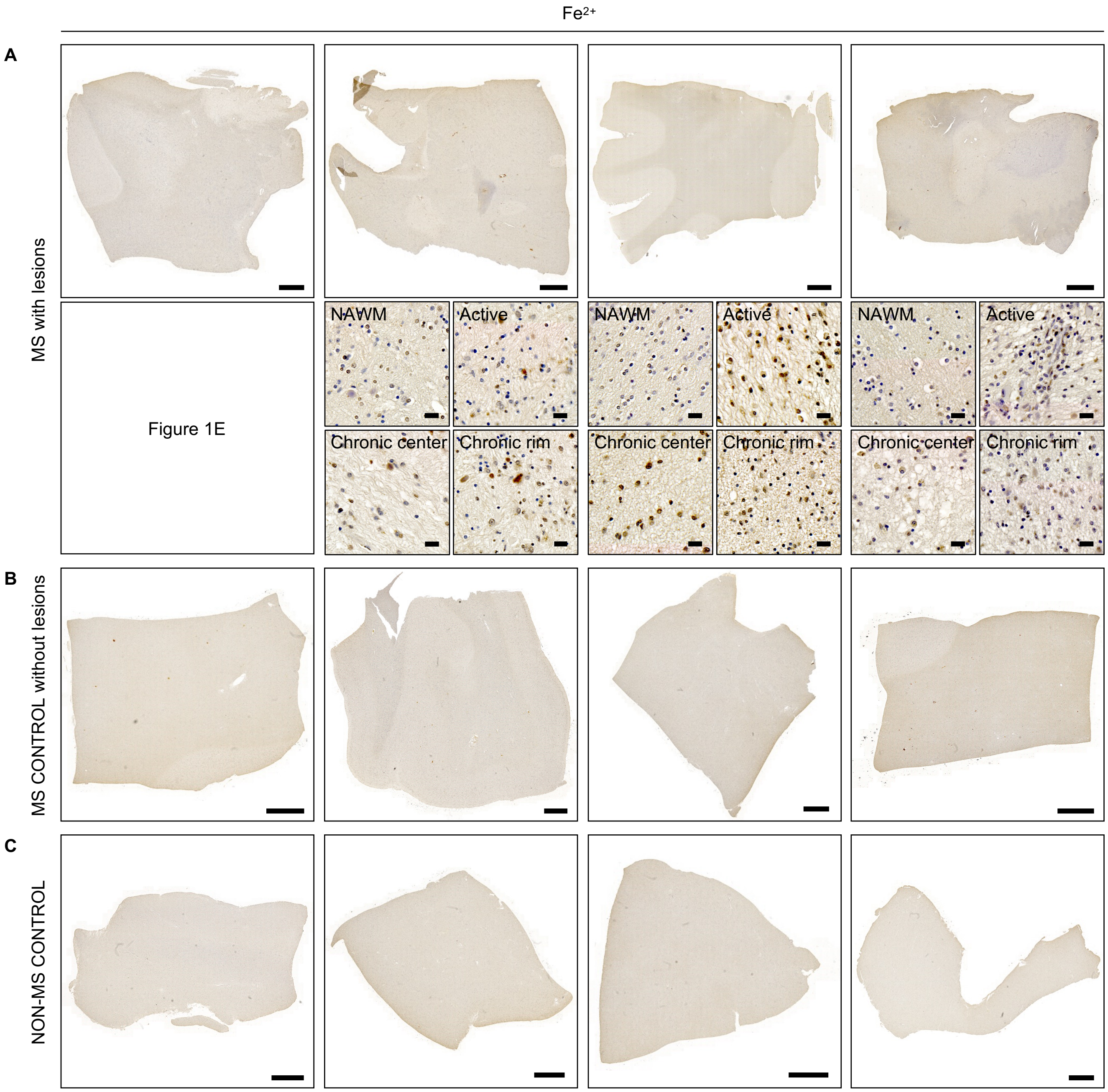

Figure S5: Paraffin-embedded sections from (**A**) lesion-containing MS, (**B**) non-lesion containing MS and (**C**) non-demented control brain tissue were stained for ferrous iron (Fe^{2+}). n=4/group. Scale bars, 2 mm and 20 μm (zoom). Normal appearing white matter (NAWM), active and chronic regions of interest are indicated in Fig. S1 by blue, orange and green dotted line, respectively.

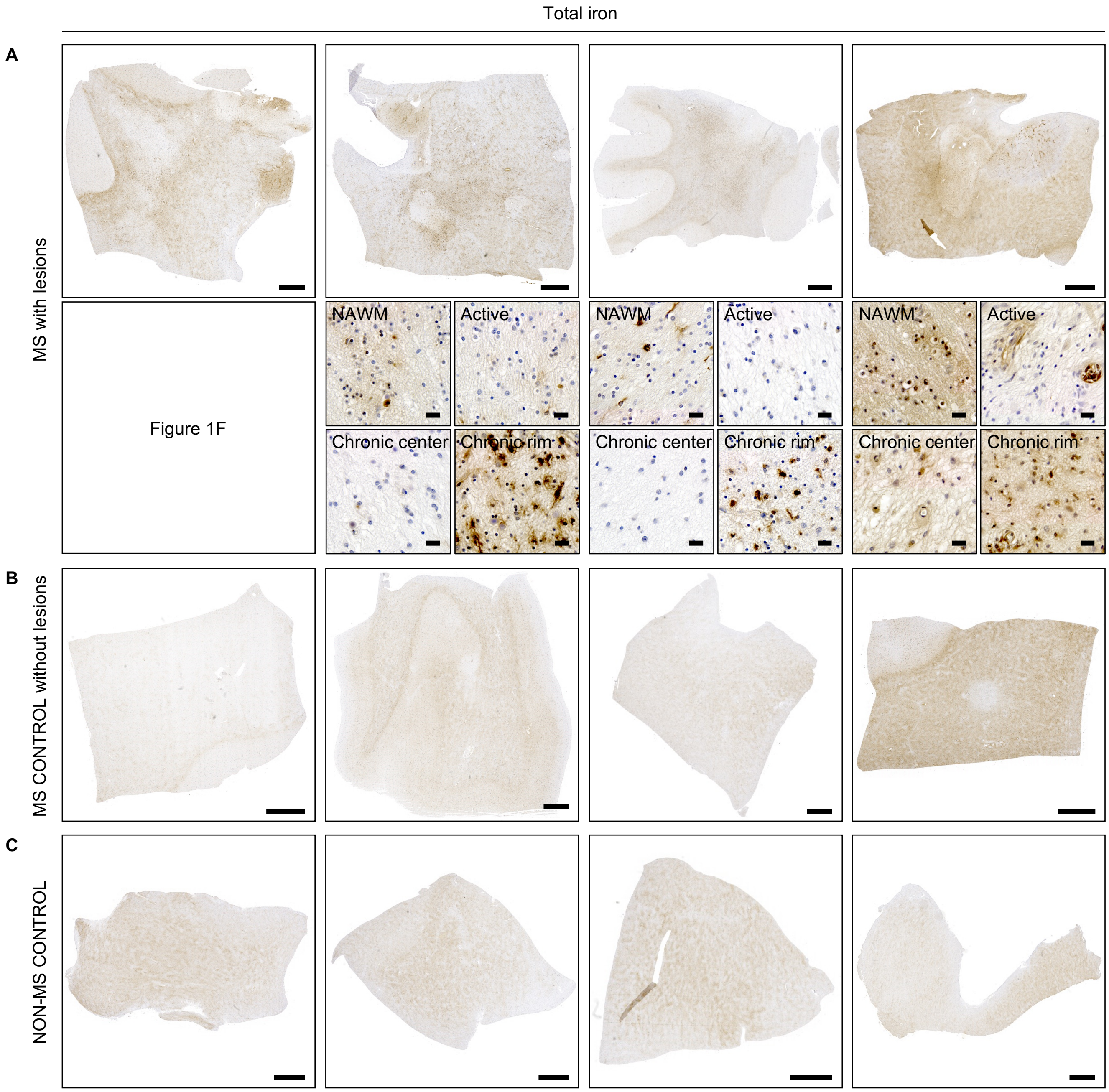

Figure S6. Paraffin-embedded sections from (**A**) lesion-containing MS, (**B**) non-lesion containing MS and (**C**) non-demented control brain tissue were stained for total non-heme iron. n=4/group. Scale bars, 2 mm and 20 μm (zoom). Normal appearing white matter (NAWM), active and chronic regions of interest are indicated in Fig. S1 by blue, orange and green dotted line, respectively.

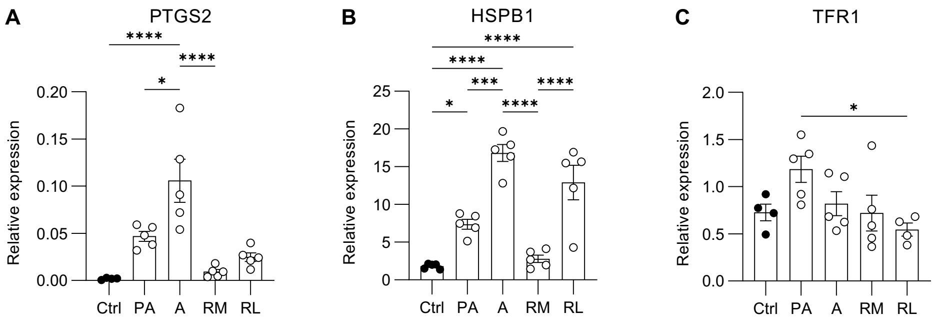

Figure S7. Relative mRNA expression levels of prostaglandin-endoperoxide synthase 2 (PTGS2) (**A**), heat shock protein beta-1 (HSPB1) (**B**) and transferrin receptor 1 (TFR1) (**C**) in isolated spinal cord of control (Ctrl) (n=4-5), pre-acute (PA) (n=5), acute (A) (n=5), remitted (RM) (n=5) and relapsing (RL) (n=4-5) EAE Biozzi ABH mice. Data is represented as ratio of mRNA expression normalized to endogenous housekeeping genes and expressed as mean ± SEM. Data was analyzed using one-way ANOVA with Tukey's correction for multiple comparisons (*$p \leq 0.05$, **$p \leq 0.01$, ***$p \leq 0.001$, ****$p \leq 0.0001$).

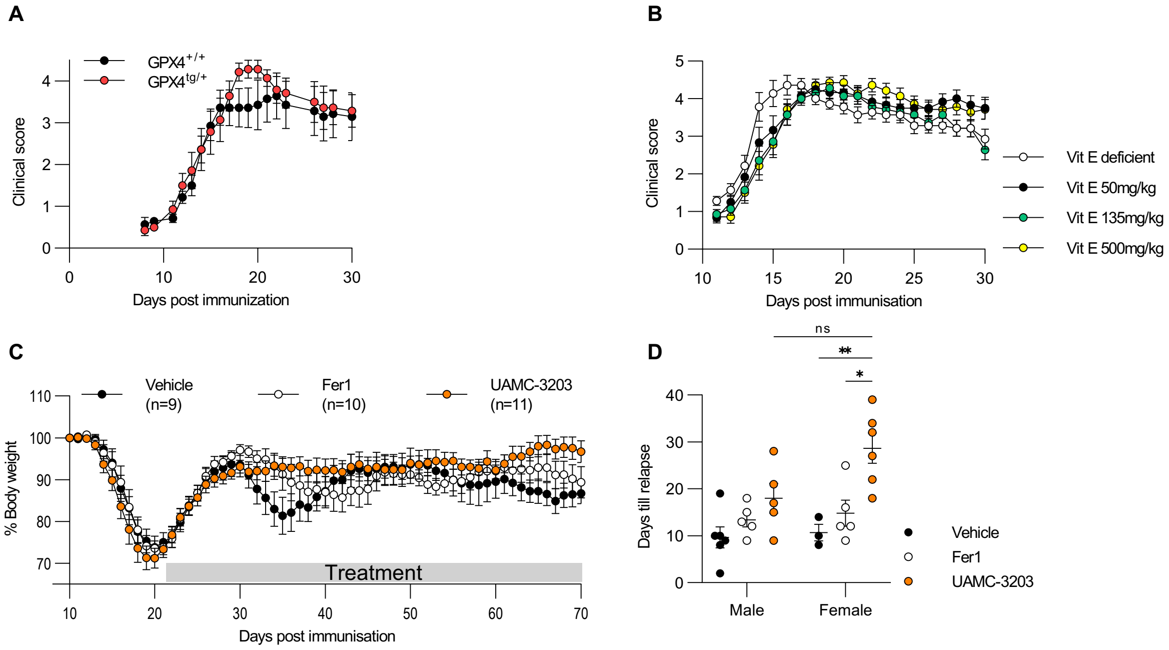

Figure S8. Clinical disease progression of EAE induced GPX4$^{+/+}$ and GPX4$^{tg/+}$ mice (**A**) and C57Bl/6N mice on adapted vitamin E diet containing <5 mg/kg vitamin E (deficient) (n=7), 50 mg/kg vitamin E (n=6), 135 mg/kg vitamin E (n=7) or 500 mg/kg vitamin E (n=7) (**B**). **C,** Loss of body weight in EAE induced Biozzi ABH mouse treated with vehicle (n=9), Fer-1 (n=10) or UAMC-3203 (n=11) from score 3 in remission (indicated by grey bar), and corresponding quantification of number of days till relapse when comparing male versus female (**D**). Data is represented as mean ± SEM. Data was analyzed by REML analysis (**A-C**) or by ordinary two-way ANOVA with Tukey's correction for multiple comparisons (*p≤0.05, **p≤0.01) (**D**).
